# Effect of Dietary Intake of Advanced Glycation End Products on Metabolic Parameters and Anthropometric Measurements in Adults

**DOI:** 10.7759/cureus.86752

**Published:** 2025-06-25

**Authors:** Ayfer Beyaz Coskun

**Affiliations:** 1 Nutrition and Dietetics, Firat University, Elazig, TUR

**Keywords:** advanced glycation end products, anthropometric measurement, cardiovascular disease, metabolic parameters, metabolic syndrome

## Abstract

Background

Advanced glycation end products (AGEs) are exogenously taken up and endogenously secreted. The amount of intake and the level of effect can be affected by dietary interventions and some lifestyle factors.

Methodology

This study was conducted with 920 adults aged 18-64 years. The physical activity status of the participants was questioned, anthropometric measurements were taken, and blood biochemical findings were recorded to calculate metabolic syndrome and cardiovascular disease risk. Daily consumption of carboxymethyl lysine, an important type of AGEs, was calculated with a validated semi-quantitative food frequency questionnaire and classified as high, moderate, and low intake levels.

Results

Those with high AGE consumption ate significantly more at lunch and dinner. It was found that 446 (73.8%) women consumed significantly more AGE (*P* = 0.003). Additionally, the majority of individuals with high AGE consumption (628, 74.4%) engaged in significantly more inadequate physical activity (*P* = 0.007). The risk of metabolic syndrome was found to be significantly higher in those with high AGE consumption than in those with moderate consumption (*P *= 0.029). AGE consumption was inversely proportional to high-density lipoprotein (HDL) cholesterol (HDL-c) (β (standard error [SE]) = -0.003 (0.001), *P* = 0.019). AGE consumption amounts were found to be directly proportional to hip circumference (β (SE) = 0.005 (0.001), *P* = 0.000), fasting blood glucose (β (SE) = 0.001 (0.000), *P* = 0.008), total cholesterol (β (SE) = 0.001 (0.000), *P* = 0.009), LDL-cholesterol (β (SE) = 0.002 (0.000), *P* = 0.000), and diastolic blood pressure (β (SE) = 0.004 (0.002), *P *= 0.005).

Conclusions

Dietary AGE intake affects anthropometric measurements and biochemical findings. Future studies can be planned as interventions that will reduce dietary AGE intake or its effects on the body and observe its metabolic effects in the long term.

## Introduction

Advanced glycation end products (AGEs) belong to a heterogeneous, complex group of compounds formed exogenously or endogenously from different mechanisms and various precursors. In general, AGEs are formed non-enzymatically between the carbonyl groups of reducing sugars and the free amine groups of proteins, nucleic acids, or lipids, followed by the formation of stable, irreversible end products [1]. These endogenously formed AGEs, together with ingested dietary AGEs (dAGEs), contribute to further protein modifications and oxidative stress, resulting in activation of NF-κB and, consequently, enhanced production of inflammatory cytokines [2].

Factors affecting the AGE content of foods depend on their protein, fat, and sugar content and the type of processing and cooking methods used, especially the preparation temperature and duration. Prolonged high temperatures, such as those used in the processing of some dairy products, and cooking techniques, such as roasting and frying, increase the production of dAGEs [3,4]. In addition, protein-rich foods, fat-rich foods, frying products, and animal products contain high levels of AGEs. Low-fat products, high-carbohydrate products, products cooked at low temperatures, and raw products contain low levels of AGEs [5]. Exogenous AGEs can be found in a wide variety of products, such as cookies, biscuits, as well as bread, peanut butter, or processed meat [6]. High AGE content is also present in cereals containing large amounts of fructose, ice cream, and soft drinks containing corn syrup [7,8]. The highest levels of exogenous AGEs in foods of animal origin are found in beef and matured cheeses, such as Parmesan cheese. This group of products is followed by poultry, pork, fish, and eggs [9]. High-fat spreads such as butter, margarine, cream cheese, and mayonnaise also contain large amounts of AGEs due to the heat extraction and refining processes involved in their production [10]. In addition, some of the highest AGE values ​​were observed in canned meat, cereal preparations processed at high temperatures, and roasted coffee [4].

The degree of AGE formation is associated with cardiovascular diseases (CVDs) [11,12]; diabetes, neuropathy, and retinopathy [13,14]; neurodegenerative diseases such as Alzheimer’s disease [15,16], Parkinson’s disease [17,18], and amyotrophic lateral sclerosis [19,20]; and the progression of traumatic brain injury [21]. AGEs from processed foods are produced during dry heat technology [22], which explains the high AGE levels in ready-made cookies, biscuits, or chips [23]. However, more and more children and infants are consuming these high AGE products, which is alarming because of the increased likelihood of developing diabetes, renal failure, heart disease, and dementia due to the excessive load of exogenous AGE [24,25].

The chemical structures of AGEs and other Maillard reaction products formed during processing (mostly during thermal processing) in foods are more complex than those formed under physiological conditions [26]. Due to this chemical diversity and other issues, it has been difficult to estimate the contribution of dAGEs. This study aimed to investigate the effect of dAGEs consumption on anthropometric measurements and biochemical findings.

## Materials and methods

Study population

This study was conducted voluntarily with individuals aged 19 to 64 years. Before the study, participants were informed about the purpose and method, and then a consent form was signed by the individuals. Ethical approval for this study was obtained from the Fırat University Non-Interventional Research Ethics Committee (Approval No. 2024/04-26).

Data collection

The study was conducted with a total of 920 individuals aged 19 to 64 who applied to family health centers. A sample size calculation was made for the study using data from a similar reference study conducted by Angoorani et al. [27] Using these data, a power analysis was performed to determine the sample size required for the study, targeting a similar effect size (Cohen’s *f* ≈ 0.25), with an alpha level of 0.05 and a power of 0.95. The analysis showed that a total sample size of approximately 880 participants would be sufficient to achieve robust statistical power to detect significant differences between groups [27]. Therefore, with a sample size of approximately 880 participants, the study was designed to provide reliable and comprehensive findings in the AGE and biochemical findings quadrants. Participants were included in the study voluntarily. Individuals who had previously been diagnosed with any psychiatric disease by a physician and whose patient records could not be accessed within the recommended time frame were not included in the study.

Classification of obesity

According to the World Health Organization classification, those with a body mass index (BMI) value between 25-29.9 are classified as overweight, those between 30 and 34.9 are classified as Class I obesity, those between 35 and 39.9 are classified as Class II obesity, and those with a BMI value ≥ 40 are classified as Class III obesity [28].

Anthropometry and blood pressure measurements

Individuals' heights were measured, and their body weights were recorded. BMI was calculated using body weight and height. Participants' waist, neck, and hip circumferences were also measured with a non-stretchable tape measure. Systolic and diastolic blood pressures were measured while they were at rest for 10 minutes.

Biochemical parameters

Participants' high-density lipoprotein cholesterol (HDL-c), low-density lipoprotein cholesterol (LDL-c), total cholesterol, and fasting blood glucose (FBG) values ​​were obtained from the patients' biochemical parameter results in the last week, and no extra blood was taken from the patients.

Determining physical activity level

A three-question short form used by Marshall et al. was applied to evaluate the physical activity status of individuals. The first question asks about regular physical activity status. In the second question, “3 or more activities per week” is evaluated as 4 points, “1-2 times per week” as 2 points, “never” as 0 points; in the third question, “5 or more activities per week” is evaluated as 4 points, “3-4 times per week” as 2 points, “1-2 times per week” as 1 point, “never” as 0 points. If the score obtained by adding the second and third questions is ≥4, it indicates that the individuals are doing sufficient physical activity [29].

Determination of metabolic syndrome risk status

The diagnostic criteria for metabolic syndrome were established according to the National Cholesterol Education Program (NCEP) Adult Treatment Panel III. Risk factors are abdominal obesity (>102 cm in men, >88 cm in women), hypertriglyceridemia (≥150 mg/dL), low HDL-c (<40 mg/dL in men, <50 mg/dL in women), blood pressure ≥130/85, and FBG >110 mg/dL. The presence of metabolic syndrome was determined in the presence of at least three of the risk factors [30].

Determination of the Framingham risk score

The Framingham risk score, developed in North America and updated in Canada and used to calculate CVD risk [31,32], has been proven valid in different countries and is also recommended by the Turkish Cardiology Association [33,34]. The Framingham risk score evaluates the susceptibility of individuals to CVD according to age, gender, smoking status, lipid profile, blood pressure, and diabetes [35].

Framingham risk scores of individuals were calculated using the table published on the official website of the Turkish Cardiology Association. Individuals' age, smoking, gender, diabetes diagnosis, LDL-c and HDL-c values, and systolic and diastolic blood pressures were recorded in the system. Those with a 10-year CVD risk of 20% and above were grouped as high risk, those between 10-19% as medium risk, and those below 10% as low risk [34].

Determination of the AGE consumption amount

In the food database published by Uribarri et al., which includes approximately 500 food items, carboxymethyllysine (CML) was used as a representative of AGEs detected by enzyme-linked immunosorbent assay (ELISA) [9]. Using these food items, individuals were questioned about their consumption frequency in the last three months and the amounts consumed at one time, and recorded. Daily consumption amounts were obtained. The average daily dAGEs amount was determined by comparing it with the CML amount in 100 g of food. Then, those who consumed 4 x 106 KU were categorized as low AGE intake; those who consumed 4.1 to 15 x 106 KU AGE intake were categorized as moderate; and those who consumed AGE above 15 x 106 KU were categorized as high AGE intake [36].

Statistical analysis

In the evaluation of the data obtained from the study, IBM SPSS Statistics 26.0 (IBM Corp., Armonk, NY) statistical package program and AMOS programs were used. Groups were created according to the AGE consumption status of the participants, and tests were applied to compare the relevant parameters via the SPSS 26.0 program. Using descriptive data, number (*n*) and percentage (%) data were used, and the analysis of variance (ANOVA) test (*F*-table value) method was applied in the comparison of the independent three-group measurement values. For the pairwise comparisons of the variables with significant differences for three or more groups, the homogeneity of the variances was taken into consideration, and the *Tukey* test statistics were used in the homogeneous distribution of the variances. Independent t-test and Pearson’s chi-squared analysis were performed to evaluate the general characteristics, lifestyle habits, and compliance with the Framingham risk classification according to the AGE consumption categories. Regression analysis was performed for the total AGE intake amount and BMI, and metabolic syndrome parameters. Additionally, the AMOS program was used for structural equation modeling and Path analysis between the AGE consumption amount, BMI, and its relationship with metabolic syndrome parameters. Goodness-of-fit values, confidence intervals, standardized β coefficients, standard errors, and p values ​​are reported for modeling in AMOS. In all statistical analyses, *P *< 0.05 was accepted as the significance level.

## Results

This study was conducted with a total of 920 individuals, including 316 males (34.3%) and 604 females (65.7%), with a mean age of 40.0 ± 13.4 years. The average dAGE intake of the individuals participating in the study was 124.069 ± 95.443 kU/day. It was found that 0.2% of participants consumed low amounts, 22.6% moderate amounts, and 77.2% high amounts of AGE. Individuals with high AGE consumption were found to consume significantly more lunch and night snacks compared to those with moderate consumption (Table 1).

**Table 1:** Meal consumption status of individuals according to AGE consumption classification. a, b, and c are upper values ​​for comparison. *One-way ANOVA test; *P *< 0.05. ANOVA, analysis of variance; AGE, advanced glycation end product

Meal consumption status	Low consumption^a^ (*n *= 2, 0.2%)	Medium consumption^b^ (*n *= 208, 22.6%)	High consumption^c^ (*n *= 710, 77.2%)	Total (*n *= 920)	*P*-value
Breakfast					
Yes	2 (100%)	190 (91.7%)	676 (95.3%)	868 (95.1%)	0.099
No	-	18 (8.3%)	34 (4.7%)	52 (4.9%)	
Lunch					
Yes	1 (50.0%)	122 (59.6%)	541 (78.6%)^a,b^	664 (77.9%)	<0.001*
No	1 (50.0%)	86 (40.4%)	169 (21.4%)	256 (22.1%)	
Dinner					
Yes	2 (100%)	201 (96.9%)	701 (98.3%)	904 (98.2%)	0.124
No	-	7 (3.1%)	9 (1.7%)	16 (1.8%)	
Mid-morning snack					
Yes	-	24 (10.9%)	112 (16.5%)	136 (16.2%)	0.265
No	2 (100%)	184 (89.1%)	598 (83.5%)	784 (83.8%)	
Afternoon snack					
Yes	1 (50.0%)	118 (57.1%)	354 (49.4%)	473 (49.7%)	0.226
No	1 (50.0%)	90 (42.9%)	356 (50.6%)	447 (50.3%)	
Midnight snack					
Yes	-	76 (39.6%)	351 (51.9%)^b^	427 (51.4%)	0.002*
No	2 (100%)	132 (60.4%)	359 (48.1%)	493 (48.6%)	

No significant difference was found between the participants' average age, smoking status, sleep duration, Framingham risk score, and BMI status according to the amount of AGE consumption. It was found that 446 (73.8%) women consumed significantly higher amounts of AGE (*P *= 0.003). It was found that the majority of individuals with high AGE consumption (628, 74.4%) did significantly more insufficient physical activity (*P *= 0.007). The risk of metabolic syndrome was found to be significantly higher in those with high AGE consumption than in those with moderate consumption (*P* = 0.029).

**Table 2:** General characteristics, lifestyle habits, and adherence to the Framingham risk classification according to AGE consumption categories. Numerical variables are presented as mean ± standard deviation (except for physical activity). Nominal variables are shown as percentage (frequency); *P*-values were derived by independent t-test and Pearson’s chi-squared, respectively. a, b, and c are upper values ​​for comparison. *P < 0.05. **P < 0.01. BMI, body mass index; F, female; AGE, advanced glycation end product; MS, metabolic syndrome

	Low consumption^a^ (*n *= 2, 0.2%)	Medium consumption^b^ (*n *= 208, 22.6%)	High consumption^c^ (*n *= 710, 77.2%)	Total (*n *= 920)	*P*-value
Gender (%F)	2 (0.4%)	156 (25.8%)	446 (73.8%)^a,b^	604 (65.7%)	0.003^*^
Age (years)	44.5 ± 14.8	43.0 ± 14.1	39.0 ± 13.1	40.0 ± 13.4	0.201
Physical activity					
Sufficient physical activity	-	6 (7.9%)	82 (92.1%)	88 (9.6%)	0.007^*^
Insufficient physical activity	2 (0.3%)	202 (25.3%)	628 (74.4%)^ b^	832 (90.4%)	
Smoking					
Yes	-	42 (18.7%)	183 (81.3%)	225 (24.5%)	0.055
No	1 (0.2%)	145 (25.4%)	425 (74.4%)	571 (62.0%)	
Former	1 (0.2%)	21 (16.9%)	102 (82.3%)	124 (13.5%)	
Sleep time					
Weekday (minute)	420.0 ± 0.0	480.0 ± 77.5	420.0 ± 178.1	440.0 ± 160.7	0.987
Weekend (minute)	480.0 ± 84.9	480.0 ± 84.6	480.0 ± 90.6	480.0 ± 89.2	0.966
MS risk status					
Yes	-	34 (15.6%)	177 (84.4%)^b^	211 (22.9%)	0.029^**^
No	2 (0.3%)	174 (24.0%)	533 (75.7%)	709 (77.1%)	
Framingham risk score	-1.0 ± 15.6	0.7 ± 8.4	0.9 ± 7.6	0.8 ± 7.8	0.894
Low	1 (0.1%)	161 (22.4%)	558 (77.5%)	720 (78.3%)	0.692
Medium	1 (0.7%)	31 (23.0%)	103 (76.3%)	135 (14.7%)	
High	-	16 (24.6%)	49 (75.4%)	65 (7.0%)	
BMI (kg/m^2^)	30.7 ± 5.7	31.7 ± 5.3	31.4 ± 5.7	31.4 ± 5.6	0.687
Underweight	-	-	5 (100.0%)	5 (0.6%)	
Normal weight	-	6 (10.9%)	49 (89.1%)	55 (6.0%)	
Overweight	1 (0.3%)	83 (24.2%)	259 (75.5%)	343 (37.3%)	0.694
Obese class I	1 (0.3%)	67 (23.3%)	220 (76.4%)	288 (31.3%)	
Obese class II	-	38 (22.9%)	128 (77.1%)	166 (18.0%)	
Obese class III	-	14 (22.6%)	48 (77.4%)	62 (6.8%)	

The relationship between the amount of AGEs consumed by individuals and biochemical and anthropometric measurements was shown using linear regression (Table 3). AGE consumption was inversely proportional to HDL-c (β (standard error [SE]) = -0.003 (0.001), *P* = 0.019) and explained 0.6% of HDL-c. AGE consumption was directly proportional to hip circumference (β (SE) = 0.005 (0.001), *P* < 0.001), FBG (β (SE) = 0.001 (<0.001), *P *= 0.008), total cholesterol (β (SE) = 0.001 (<0.001), *P *= 0.009), LDL-c (β (SE) = 0.002 (<0.001), *P *< 0.001), and diastolic blood pressure (β (SE) = 0.004 (0.002), *P *= 0.005). It explained 2.1% of hip circumference, 0.8% of FBG, 0.7% of total cholesterol, 2.2% of LDL-c, and 0.9% of diastolic blood pressure. No relationship was found between other anthropometric measurements and systolic blood pressure and AGE consumption.

**Table 3:** Beta coefficients (standard error) of AGE classification according to each standard deviation increase in anthropometric and biochemical measurements from linear regression analysis. Beta coefficient, standard error, 95% confidence interval (CI), *R*^2^, and *P*-value calculated from linear regression analysis. *P < 0.05. BMI, body mass index; FBG, fasting blood glucose; HDL-c, high-density lipoprotein-cholesterol; LDL-c, low-density lipoprotein-cholesterol; WC, waist circumference; NC, neck circumference; HC, hip circumference; Total-c, cholesterol; SBP, systolic blood pressure; DBP, diastolic blood pressure; SE, standard error

Dependent variable	β (SE)	95% CI (lower-upper limit)	*R*^2^	*P*-value
BMI (kg/m^2^)	-0.002 (0.003)	-0.007 to 0.003	0.001	0.428
NC (cm)	0.003 (0.003)	-0.003 to 0.010	0.001	0.313
WC (cm)	-0.002 (0.001)	-0.004 to 0.000	0.003	0.081
HC (cm)	0.005 (0.001)	0.001 to 0.003	0.021	<0.001*
FBG (mg/dL)	0.001 (<0.001)	0.000 to 0.001	0.008	0.008*
Total-c (mg/dL)	0.001 (<0.001)	0.000 to 0.001	0.007	0.009*
HDL-c (mg/dL)	-0.003 (0.001)	-0.006 to -0.001	0.006	0.019*
LDL-c (mg/dL)	0.002 (<0.001)	0.001 to 0.002	0.022	<0.001*
SBP (mmHg)	0.000 (<0.001)	0.000 to 0.001	0.002	0.217
DBP (mmHg)	0.004 (0.002)	0.001 to 0.007	0.009	0.005*

In Table 4, AGE consumption and BMI, FBG, HDL-c, and LDL-c values exhibit a moderate and significant relationship (*R* = 0.142, *R*² = 0.020, *P* < 0.001), with AGE consumption accounting for 2% of the total variance. A 2% change in the dependent variable is explained by the independent variables included in the model (BMI, FBG, HDL-c, LDL-c). When the t-test results regarding the significance of the regression coefficients are examined, it is observed that AGE consumption amount has a significant and positive relationship with BMI, FBG, and LDL-c, and a significant and negative relationship with HDL-c.

**Table 4:** Results of regression analysis on total AGE intake and BMI and metabolic syndrome parameters. *R* = 0.142, *R*^2^ = 0.020; *F* = 286208.563; *P* < 0.001. Dependent variable: AGE consumption amount *Linear regression analysis; *P* < 0.05. BMI, body mass index; FBG, fasting blood glucose; HDL-c, high-density lipoprotein-cholesterol; LDL-c, low-density lipoprotein-cholesterol; SE, standard error; AGE, advanced glycation end product

Variable	B	SE	β	t	*P-*value
Sabit	112835.803	100.612	-	1121.495	<0.001*
BMI (kg/m^2^)	587.283	2.232	0.035	263.137	<0.001*
FBG (mg/dL)	4.537	0.242	0.003	18.751	<0.001*
HDL-c (mg/dL)	-387.058	1.418	-0.036	-272.931	<0.001*
LDL-c (mg/dL)	313.892	0.340	0.142	922.095	<0.001*

## Discussion

AGEs are produced by glycation in long-lived extracellular proteins or cells. Protein glycation, collectively called the Maillard reaction, is present in fluids and all tissues where significant amounts of fructose, glucose, or more reactive dicarbonyls react with proteins [37]. Based on a rodent study, Koschinsky et al. estimated that 10% of orally ingested AGEs are absorbed, of which only 30% is excreted in the urine [38]. This initial study suggested that a significant portion of dAGEs is likely to be retained in the body. Although some reactive dicarbonyls have been found in significant amounts in foods [39], recent data suggest that they are unlikely to reach the circulation [40]. For example, a study on methylglyoxal, one of the most studied intermediates, has shown that dietary methylglyoxal is rapidly degraded in the gastrointestinal tract [40].

Studies based on exposure to CML, one of the end products of protein glycation, or its ¹³C isotopologue, have shown that chronic ingestion of this AGE by mice leads to increased accumulation in the kidneys, intestines, lungs, brain, heart, blood vessels, and other tissues [41,42]. Apart from animal studies, some observational or interventional human studies have been conducted to confirm the contribution of exogenous AGEs to the in vivo AGE pool [43]. For example, an increased serum AGE level was observed after ingestion of a meal consisting of glycated egg whites with glucose [38]. The study findings should be interpreted with some limitations, as there is no consensus yet among the different groups studying dAGEs on which molecule should be considered the most representative. However, in the food database published by Uribarri et al., which includes approximately 500 food items, CML was used as a representative of AGEs detected by ELISA and was also used in this study [9].

Increased physical activity prevents the accumulation of dAGEs and attenuates their adverse metabolic effects, and exercise reduces oxidative stress and inflammation exacerbated by AGEs [44]. Exercise enhances the effects of a low-AGE diet and reduces serum AGE levels [45]. Regular exercise may reduce hyperglycemia and thus endogenous AGE formation by increasing insulin sensitivity [46]. This study found that the majority of individuals with high AGE intake engaged in significantly more inadequate physical activity. Both high dAGE intake and inadequate physical activity increase the risks. It appears important to combine dietary changes with exercise to reduce AGE-related risks.

Toxins in cigarette smoke accelerate protein glycation by increasing oxidative stress and lead to the accumulation of AGEs [47]. Cigarette smoke induces the formation of AGEs and reduces the soluble cell-bound receptor, leading to the development of atherosclerosis and related stroke, coronary heart disease, and peripheral vascular disease [48]. In this study, no significant difference was found among smokers according to the AGE consumption category. Since this study did not examine the AGE effect levels in the body of smokers, comparison with the literature cannot be made. However, the combined effect of exogenous and endogenous AGEs may accelerate oxidative stress, and quitting smoking may be critical in populations with high AGE exposure.

AGEs can negatively affect sleep health, and reducing AGE intake can facilitate the prevention and improvement of sleep disorders [49]. In this study, no significant difference was found between sleep times according to different levels of AGE intake. Since the extent to which AGE intake affects sleep health or triggers sleep disorders was not questioned, more studies are needed on this subject.

Diets containing high AGEs (fried meats and processed foods) are generally high in calories and saturated fat. Such eating habits are directly related to increases in waist circumference and BMI. The fact that AGEs increase insulin resistance can trigger obesity by facilitating the accumulation of adipose tissue [50]. In a study, the amount of dAGEs taken per kcal was calculated using a 24-hour food consumption record and classified as low, medium, and high in 80 participants. It was found that women with low dAGEs had approximately 5% less gynoid fat than those with high and medium dAGEs [51]. In this study, it was found that those who consumed high AGEs consumed significantly more lunch and night snacks. Food consumption in the late hours is a factor that increases fat accumulation. In addition, AGE consumption amounts were found to be directly proportional to hip circumference. Therefore, in this study, similar to the literature, it was found that as AGE consumption increases, there may be an increase in the lower body area, namely the hip circumference, which may lead to gynoid fattening.

Dietary AGEs can change both metabolic and hormonal parameters of affected women without BMI changes [52]. Studies in healthy humans show that dAGEs are directly associated with circulating AGEs and markers of oxidative stress, such as CML and methylglyoxal. In a cohort of healthy adults from the New York City area, mean dAGEs intake was found to be 14,700 ± 680 AGE kU/day [53]. A safe and optimal intake of dAGEs for disease prevention purposes has not yet been determined. However, in animal studies, reducing dAGEs to 50% of normal intake has been associated with reduced levels of oxidative stress, less deterioration of insulin sensitivity and kidney function with age, and longer lifespan. Low dAGEs intake has also been shown to extend lifespan, as has energy restriction in mice [54]. In a study investigating whether the AGE content of a single meal affects postprandial appetite and markers of inflammation, endothelial activation, and oxidative stress in healthy obese individuals, it was shown that AGEs can affect postprandial ghrelin, oxidative stress, and glucose responses [55]. Taylor et al. suggested that a low-glycemic index (GI) diet may help limit spikes in blood glucose and thereby reduce AGE levels in vivo [56], similar to how good long-term glycemic control lowers AGE levels in patients with type 1 diabetes [57]. This group found that 11-month-old mice fed a high-GI diet had impaired glucose tolerance compared to mice fed a low-GI diet. They also found that these mice had at least three times more AGE accumulation in the retina, liver, and brain [58]. In this study, AGE consumption was directly proportional to FBG. Increased AGE consumption may increase the risk of both FBG and metabolic syndrome.

The AGE-RAGE (receptor for AGE) axis is associated with arterial stiffness and hypertension. Studies suggest that inhibition of AGE formation, reduction of AGE consumption, blockade of the AGE-RAGE interaction, suppression of RAGE expression, and exogenous administration of soluble RAGE may be novel therapeutic strategies for treating arterial stiffness and hypertension [59]. A study of 5,848 adults determined daily CML consumption using a semiquantitative food frequency questionnaire. Those with high AGE intake were found to have a higher risk of abdominal obesity and hypertriglyceridemia compared with those with low intake [27]. Another study found that consuming a diet high or low in AGEs for six weeks in healthy middle-aged and older adults did not affect endothelial function and inflammatory mediators, two precursors of cardiovascular disease [60]. In this study, AGE intake was directly proportional to total cholesterol, LDL-c, and diastolic blood pressure, and inversely proportional to HDL-c. No significant difference was found in Framingham risk score, but it was observed that it may affect the cardiovascular disease components.

Limitations of the study

This study is observational in nature, which limits the ability to establish causal relationships. A 24-hour retrospective recall of dietary intake could have also provided an opportunity to compare daily energy and nutrient intakes and glycemic load. In addition, inflammatory markers could have been questioned for comparison.

## Conclusions

Dietary AGE intake affects both anthropometric measurements and biochemical findings, and the risk of metabolic syndrome. Interventions that reduce dAGEs (e.g., low-AGE diets) may show promising results in reducing cardiovascular risk markers, and AGEs may be among the modifiable factors in hypertension management.

Heterogeneity in AGE assessment methods (e.g., diet diaries and serum CML) complicates comparisons. Future randomized controlled trials may help determine causality and clarify interactions between AGEs and lifestyle factors across diverse populations.

## References

[1] Twarda-Clapa A, Olczak A, Białkowska AM, Koziołkiewicz M (2022). Advanced glycation end-products (AGEs): Formation, chemistry, classification, receptors, and diseases related to AGEs. Cells.

[2] Reddy VP, Aryal P, Darkwah EK (2022). Advanced glycation end products in health and disease. Microorganisms.

[3] Lund MN, Ray CA (2017). Control of Maillard reactions in foods: Strategies and chemical mechanisms. J Agric Food Chem.

[4] Scheijen JL, Clevers E, Engelen L, Dagnelie PC, Brouns F, Stehouwer CD, Schalkwijk CG (2016). Analysis of advanced glycation endproducts in selected food items by ultra-performance liquid chromatography tandem mass spectrometry: Presentation of a dietary AGE database. Food Chem.

[5] Zawada A, Machowiak A, Rychter AM, Ratajczak AE, Szymczak-Tomczak A, Dobrowolska A, Krela-Kaźmierczak I (2022). Accumulation of advanced glycation end-products in the body and dietary habits. Nutrients.

[6] Assar SH, Moloney C, Lima M, Magee R, Ames JM (2009). Determination of Nepsilon-(carboxymethyl)lysine in food systems by ultra performance liquid chromatography-mass spectrometry. Amino Acids.

[7] Delgado-Andrade C, Rufián-Henares JA, Morales FJ (2006). Study on fluorescence of Maillard reaction compounds in breakfast cereals. Mol Nutr Food Res.

[8] Tan AL, Sourris KC, Harcourt BE (2010). Disparate effects on renal and oxidative parameters following RAGE deletion, AGE accumulation inhibition, or dietary AGE control in experimental diabetic nephropathy. Am J Physiol Renal Physiol.

[9] Uribarri J, Woodruff S, Goodman S (2010). Advanced glycation end products in foods and a practical guide to their reduction in the diet. J Am Diet Assoc.

[10] Vlassara H, Uribarri J (2004). Glycoxidation and diabetic complications: modern lessons and a warning?. Rev Endocr Metab Disord.

[11] Stratmann B (2022). Dicarbonyl stress in diabetic vascular disease. Int J Mol Sci.

[12] Pinto RS, Ferreira GS, Silvestre GC (2022). Plasma advanced glycation end products and soluble receptor for advanced glycation end products as indicators of sterol content in human carotid atherosclerotic plaques. Diab Vasc Dis Res.

[13] Fotheringham AK, Gallo LA, Borg DJ, Forbes JM (2022). Advanced glycation end products (AGEs) and chronic kidney disease: does the modern diet AGE the kidney?. Nutrients.

[14] Forbes JM, Coughlan MT, Cooper ME (2008). Oxidative stress as a major culprit in kidney disease in diabetes. Diabetes.

[15] Haddad M, Perrotte M, Landri S, Lepage A, Fülöp T, Ramassamy C (2019). Circulating and extracellular vesicles levels of N-(1-Carboxymethyl)-L-Lysine (CML) differentiate early to moderate Alzheimer’s disease. J Alzheimers Dis.

[16] Dei R, Takeda A, Niwa H (2002). Lipid peroxidation and advanced glycation end products in the brain in normal aging and in Alzheimer's disease. Acta Neuropathol.

[17] Sasaki NA, Sonnet P (2021). A novel multi-target strategy to attenuate the progression of Parkinson's disease by diamine hybrid AGE/ALE inhibitor. Future Med Chem.

[18] Chrysanthou M, Miro Estruch I, Rietjens IM, Wichers HJ, Hoppenbrouwers T (2022). In vitro methodologies to study the role of advanced glycation end products (AGEs) in neurodegeneration. Nutrients.

[19] Juranek JK, Daffu GK, Wojtkiewicz J, Lacomis D, Kofler J, Schmidt AM (2015). Receptor for advanced glycation end products and its inflammatory ligands are upregulated in amyotrophic lateral sclerosis. Front Cell Neurosci.

[20] MacLean M, Juranek J, Cuddapah S (2021). Microglia RAGE exacerbates the progression of neurodegeneration within the SOD1(G93A) murine model of amyotrophic lateral sclerosis in a sex-dependent manner. J Neuroinflammation.

[21] Saglam E, Zırh S, Aktas CC, Muftuoglu SF, Bilginer B (2021). Papaverine provides neuroprotection by suppressing neuroinflammation and apoptosis in the traumatic brain injury via RAGE- NF-B pathway. J Neuroimmunol.

[22] Snelson M, Coughlan MT (2019). Dietary advanced glycation end products: digestion, metabolism and modulation of gut microbial ecology. Nutrients.

[23] Sharma C, Kaur A, Thind SS, Singh B, Raina S (2015). Advanced glycation end-products (AGEs): an emerging concern for processed food industries. J Food Sci Technol.

[24] Kutlu T (2016). Dietary glycotoxins and infant formulas. Turk Pediatri Ars.

[25] Teissier T, Quersin V, Gnemmi V (2019). Knockout of receptor for advanced glycation end-products attenuates age-related renal lesions. Aging Cell.

[26] Tessier FJ, Niquet C (2007). The metabolic, nutritional and toxicological consequences of ingested dietary Maillard reaction products: a literature review. J Soc Biol.

[27] Angoorani P, Ejtahed HS, Mirmiran P, Mirzaei S, Azizi F (2016). Dietary consumption of advanced glycation end products and risk of metabolic syndrome. Int J Food Sci Nutr.

[28] Pi-Sunyer FX (2000). Obesity: criteria and classification. Proc Nutr Soc.

[29] Marshall AL, Smith BJ, Bauman AE, Kaur S (2005). Reliability and validity of a brief physical activity assessment for use by family doctors. Br J Sports Med.

[30] Zimmet P, Alberti G, Shaw J (2005). A new IDF worldwide definition of the metabolic syndrome: of the metabolic syndrome: the rationale and the results. Diabetes Voice.

[31] Bosomworth NJ (2011). Practical use of the Framingham risk score in primary prevention: Canadian perspective. Can Fam Physician.

[32] Wilson PW, D'Agostino RB, Levy D, Belanger AM, Silbershatz H, Kannel WB (1998). Prediction of coronary heart disease using risk factor categories. Circulation.

[33] Kasim SS, Ibrahim N, Malek S (2023). Validation of the general Framingham Risk Score (FRS), SCORE2, revised PCE and WHO CVD risk scores in an Asian population. Lancet Reg Health West Pac.

[34] Kültürsay H (2011). Kardiyovasküler hastalık riski hesaplama yöntemleri. Arch Turk Soc Cardiol.

[35] Tunstall-Pedoe H (2011). Cardiovascular risk and risk scores: ASSIGN, Framingham, QRISK and others: how to choose. Heart.

[36] Luevano-Contreras C, Chapman-Novakofski K (2010). Assessment strategy for determining dietary advanced glycation end products. J Am Diet Assoc.

[37] Chaudhuri J, Bains Y, Guha S (2018). The role of advanced glycation end products in aging and metabolic diseases: bridging association and causality. Cell Metab.

[38] Koschinsky T, He CJ, Mitsuhashi T (1997). Orally absorbed reactive glycation products (glycotoxins): an environmental risk factor in diabetic nephropathy. Proc Natl Acad Sci U S A.

[39] Degen J, Hellwig M, Henle T (2012). 1,2-dicarbonyl compounds in commonly consumed foods. J Agric Food Chem.

[40] Degen J, Vogel M, Richter D, Hellwig M, Henle T (2013). Metabolic transit of dietary methylglyoxal. J Agric Food Chem.

[41] Tessier FJ, Niquet-Léridon C, Jacolot P (2016). Quantitative assessment of organ distribution of dietary protein-bound (13) C-labeled N(ɛ) -carboxymethyllysine after a chronic oral exposure in mice. Mol Nutr Food Res.

[42] Grossin N, Auger F, Niquet-Leridon C (2015). Dietary CML-enriched protein induces functional arterial aging in a RAGE-dependent manner in mice. Mol Nutr Food Res.

[43] Tessier FJ, Birlouez-Aragon I (2012). Health effects of dietary Maillard reaction products: the results of ICARE and other studies. Amino Acids.

[44] Hooshiar SH, Esmaili H, Taherian A, Jafarnejad S (2022). Exercise, advanced glycation end products, and their effects on cardiovascular disorders: a narrative review. Heart Mind.

[45] Macías-Cervantes MH, Rodríguez-Soto JM, Uribarri J, Díaz-Cisneros FJ, Cai W, Garay-Sevilla ME (2015). Effect of an advanced glycation end product-restricted diet and exercise on metabolic parameters in adult overweight men. Nutrition.

[46] DiMenna FJ, Arad AD (2021). The acute vs. chronic effect of exercise on insulin sensitivity: nothing lasts forever. Cardiovasc Endocrinol Metab.

[47] Kwon OS, Decker ST, Zhao J (2023). The receptor for advanced glycation end products (RAGE) is involved in mitochondrial function and cigarette smoke-induced oxidative stress. Free Radic Biol Med.

[48] Prasad K, Dhar I, Caspar-Bell G (2015). Role of advanced glycation end products and its receptors in the pathogenesis of cigarette smoke-induced cardiovascular disease. Int J Angiol.

[49] Li L, Guo J, Liang X (2024). Associations of advanced glycation end products with sleep disorders in Chinese adults. Nutrients.

[50] Demirer B, Yardımcı H (2022). İleri Glikasyon Son Ürünlerinin Diyabet Komplikasyonları Üzerine Etkileri: Bir Derleme. J Nutr Diet.

[51] Mendes NP, Cândido FG, Valente FX, Peluzio MD, Juvanhol LL, Alfenas RC (2024). Dietary advanced glycation end products, body composition, and anthropometric measures: a cross-sectional analysis in women with excess body weight. Nutr Metab Cardiovasc Dis.

[52] Tantalaki E, Piperi C, Livadas S (2014). Impact of dietary modification of advanced glycation end products (AGEs) on the hormonal and metabolic profile of women with polycystic ovary syndrome (PCOS). Hormones (Athens).

[53] Uribarri J, Cai W, Peppa M, Goodman S, Ferrucci L, Striker G, Vlassara H (2007). Circulating glycotoxins and dietary advanced glycation endproducts: two links to inflammatory response, oxidative stress, and aging. J Gerontol A Biol Sci Med Sci.

[54] Cai W, He JC, Zhu L, Chen X, Zheng F, Striker GE, Vlassara H (2008). Oral glycotoxins determine the effects of calorie restriction on oxidant stress, age-related diseases, and lifespan. Am J Pathol.

[55] Poulsen MW, Bak MJ, Andersen JM (2014). Effect of dietary advanced glycation end products on postprandial appetite, inflammation, and endothelial activation in healthy overweight individuals. Eur J Nutr.

[56] Whitcomb EA, Chiu CJ, Taylor A (2015). Dietary glycemia as a determinant of health and longevity. Mol Aspects Med.

[57] Kostolanská J, Jakus V, Barák L (2009). HbA1c and serum levels of advanced glycation and oxidation protein products in poorly and well controlled children and adolescents with type 1 diabetes mellitus. J Pediatr Endocrinol Metab.

[58] Uchiki T, Weikel KA, Jiao W (2012). Glycation-altered proteolysis as a pathobiologic mechanism that links dietary glycemic index, aging, and age-related disease (in nondiabetics). Aging Cell.

[59] Prasad K, Mishra M (2017). Do advanced glycation end products and its receptor play a role in pathophysiology of hypertension?. Int J Angiol.

[60] Semba RD, Gebauer SK, Baer DJ (2014). Dietary intake of advanced glycation end products did not affect endothelial function and inflammation in healthy adults in a randomized controlled trial. J Nutr.

